# Discordant inflammation and pain in early and established rheumatoid arthritis: Latent Class Analysis of Early Rheumatoid Arthritis Network and British Society for Rheumatology Biologics Register data

**DOI:** 10.1186/s13075-016-1186-8

**Published:** 2016-12-13

**Authors:** Daniel F. McWilliams, Eamonn Ferguson, Adam Young, Patrick D. W. Kiely, David A. Walsh

**Affiliations:** 1 Division of Rheumatology, Orthopaedics and Dermatology, School of Medicine, University of Nottingham, City Hospital, Nottingham, NG5 1PB UK; 2 Arthritis Research UK Pain Centre, University of Nottingham, Nottingham, UK; 3 School of Psychology, University of Nottingham, Nottingham, UK; 4 West Hertfordshire Hospitals NHS Trust, St. Albans, UK; 5 St. George’s Healthcare NHS Trust, London, UK

**Keywords:** Rheumatoid arthritis, Pain, Inflammation, Latent class analysis

## Abstract

**Background:**

Rheumatoid arthritis (RA) disease activity is often measured using the 28-joint Disease Activity Score (DAS28). We aimed to identify and independently verify subgroups of people with RA that may be discordant with respect to self-reported and objective disease state, with potentially different clinical needs.

**Methods:**

Data were derived from three cohorts: (1) the Early Rheumatoid Arthritis Network (ERAN) and the British Society for Rheumatology Biologics Register (BSRBR), (2) those commencing tumour necrosis factor (TNF)-α inhibitors and (3) those using non-biologic drugs. In latent class analysis, we used variables related to pain, central pain mechanisms or inflammation (pain, vitality, mental health, erythrocyte sedimentation rate, swollen joint count, tender joint count, visual analogue scale of general health). Clinically relevant outcomes were examined.

**Results:**

Five, four and four latent classes were found in the ERAN, BSRBR TNF inhibitor and non-biologic cohorts, respectively. The proportions of people assigned with >80% probability into latent classes were 76%, 58% and 72% in the ERAN, TNF inhibitor and non-biologic cohorts, respectively. The latent classes displayed either concordance between measures indicative of mild, moderate or severe disease activity; discordantly worse patient-reported measures despite less markedly elevated inflammation; or discordantly less severe patient-reported measures despite elevated inflammation. Latent classes with discordantly worse patient-reported measures represented 12%, 40% and 21% of the ERAN, TNF inhibitor and non-biologic cohorts, respectively; contained more females; and showed worse function. In those latent classes with worse scores at baseline, DAS28 and function improved over 1 year (*p* < 0.001 for all comparisons), and scores differed less at follow-up than at baseline.

**Conclusions:**

Discordant latent classes can be identified in people with RA, and these findings are robust across three cohorts with varying disease duration and activity. These findings could be used to identify a sizeable subgroup of people with RA who might gain added benefit from pain management strategies.

**Electronic supplementary material:**

The online version of this article (doi:10.1186/s13075-016-1186-8) contains supplementary material, which is available to authorized users.

## Background

Rheumatoid arthritis (RA) is an inflammatory arthritis in which chronic pain is prevalent. Inflammatory disease activity in RA is often measured using the composite 28-joint Disease Activity Score (DAS28), consisting of 28-joint swollen joint count (SJC), 28-joint tender joint count (TJC), erythrocyte sedimentation rate (ESR) and visual analogue scale (VAS) for general health (VAS-GH). These components are either patient-reported (TJC, VAS), clinician-assessed (SJC) or laboratory-measured. Patient-reported components reflect symptomatic disease, whilst other components more directly address inflammatory pathology. The discrete DAS28 components are each correlated with one another, supporting the view that DAS28 measures a single entity (RA inflammatory disease activity) [1]. However, the two patient-reported components (TJC and VAS-GH) are also strongly influenced by reported pain, which might be moderated by factors additional to inflammation, such as the processing of afferent signals by the central nervous system [2–4]. Indeed, there is now considerable evidence that psychological factors such as anxiety and vitality influence not only inflammatory and immune responses but also perception of pain [5].

TJC, VAS and the proportion of DAS28 attributable to patient-reported measures were each associated with reported pain [6, 7], and they predicted future pain in people with RA [3, 8]. Patient-reported DAS28 components in RA were also associated with widespread low pain pressure thresholds (suggesting augmented central pain processing), poorer mental health, fatigue and fibromyalgia status, independent of inflammatory disease activity [6–9]. Pain, fatigue and mental health are closely inter-related constructs because of their overlapping symptoms and shared central neurological mechanisms [8, 10–12] as well as psychological traits such as anxiety [5]. Each is recognised in people without RA who are diagnosed with fibromyalgia [12], and people with fibromyalgia might display DAS28 scores similar to those of people with active RA, predominantly owing to high TJC and VAS, and despite an absence of any joint inflammation [13]. One-fifth of people with RA also satisfied classification criteria for fibromyalgia, and they display multiple tender points at non-joint sites, widespread pain, mood disturbance and fatigue [10–12]. In these cases, augmented pain processing might contribute to pain severity, thereby worsening patient-reported DAS28 components and total DAS28 without concordant inflammation and without necessarily leading to joint damage [14, 15].

People with discordantly high patient-reported DAS28 components, fatigue and mood disturbance might represent a subgroup of people with RA who have unique clinical needs. Defining patient subgroups, being person-based rather than variable-based, can help target treatments towards those people who are most likely to benefit [5]. Treatments of some tumours are already stratified according to molecular profiles [16], and research is underway to stratify RA therapies on the basis of inflammatory mechanisms [17, 18]. Identifying patient subgroups in which outcomes are determined by factors other than inflammation might improve allocation of both immunomodulatory therapies and adjunctive pain management strategies [5].

Hierarchical clustering analysis previously defined a subgroup of individuals with established RA that was characterised by low levels of inflammation but high levels of symptoms [19]. Discrete subgroups within populations can also be identified by latent class analysis (LCA), a branch of structural equation modelling [20]. LCA has advantages over hierarchical clustering analysis in that the optimal number of classes is decided using clear fit statistics, and people are assigned to classes using probabilistic routines, allowing hypothesis testing for different models and reducing the amount of subjectivity in model choice [19]. Each person has a probability of membership in each subgroup, as opposed to an absolute assignment given by other methodologies. Previous studies of RA using LCA-related techniques include longitudinal trajectories of physical function [21] and genome analysis [22].

We hypothesised that simple-to-measure validated instruments and clinical examination measuring the current characteristics of pain and inflammation can be used to define discrete subgroups of people with RA for whom patient-reported symptoms are concordant, and other subgroups for whom patient-reported symptoms are discordantly worse than might be expected based on clinician-assessed or laboratory measures of inflammation. Concordant and discordant subgroups might have differing prognoses in the context of current care. We have undertaken hypothesis-driven LCA, cross-validated across three large samples: the Early Rheumatoid Arthritis Network (ERAN) cohort and two established RA cohorts from the British Society for Rheumatology Biologics Registers (BSRBR) either commencing tumour necrosis factor (TNF)-α inhibitors or using only non-biologic disease-modifying drugs [23]. Together these data reflect people with early or established RA and those treated with traditional or biologic disease-modifying anti-rheumatic drugs (DMARDs).

## Methods

ERAN was an inception cohort of people with early RA, all recruited at the time of first physician’s diagnosis of RA, with data collection from 2002 until 2014 [24]. Data were excluded if diagnosis changed at a later date (*n* = 36). The BSRBR cohorts recruited people with RA of any duration, and data are included in the present study where participants commenced either etanercept or adalimumab (TNF inhibitor cohort) or had physician-assessed active RA but were not commencing a biologic agent (non-biologic cohort) [25]. Most non-biologic cohort participants were commencing or changing non-biologic DMARD treatment [26] at baseline and therefore were thought by their clinicians to have active disease. (Baseline characteristics are summarised in Table 1.) Registration and enrolment in the BSRBR of new users of biologics was recommended by national guidelines at the time of recruitment. Data collection for BSRBR cohorts commenced in 2001, as described elsewhere [27]. For all three cohorts, data were collected using standardised questionnaires (Health Assessment Questionnaire [HAQ] and 36-item Short Form Health Survey [SF-36]), clinical examination, medical records and clinical report forms by the clinical team responsible for the patient’s care. DAS28 components (TJC, SJC, acute-phase response as measured by ESR, and VAS) were assessed at baseline, at 3–6 months (ERAN) or 6 months (BSRBR cohorts), and at 12-month follow-up visits. Mean DAS28, pain, disability and vitality outcomes for one or both of the cohorts during early follow-up have previously been reported for the ERAN [3, 28–30] and BSRBR [9, 31, 32] cohorts. All participants gave signed, informed consent to participate in line with the Declaration of Helsinki. The ERAN study was approved by Trent Research Ethics Committee (reference 01/4/047). The BSRBR studies were approved by North West Medical Research Ethics Committee (reference 00/8/53).

**Table 1:** Demographics and baseline variables

	ERAN	BSRBR	
		TNF inhibitor	Non-biologic
Total subjects	828	9905	2581
Age, years	57 (14)	56 (12)	60 (12)
Female sex	67%	76%	73%
BMI, kg/m^2^	27.6 (5.3)	27.0 (7.3)	27.4 (6.7)
Smoking status			
Never	39%	40%	38%
Ex-smoker	25%	38%	41%
Current	36%	22%	21%
Duration, years	0.7 (0.6)	13 (10)	10 (10)
Positive serology	60%	65%	57%
1987 ACR criteria, yes	53%	100%	100%
DAS28-ESR	4.7 (1.5)	6.6 (1.0)	5.1 (1.3)
ESR, mm/h	30 (24)	46 (29)	34 (24)
SJC, 0–28	6 (5)	11 (6)	6 (5)
TJC, 0–28	7 (7)	16 (7)	8 (7)
VAS, 0–100	44 (25)	72 (20)	54 (24)
HAQ, 0–3	1.1 (0.8)	2.0 (0.6)	1.5 (0.8)
SF-36			
Physical function	30 (15)	16 (11)	24 (11)
Bodily pain	33 (11)	25 (7)	31 (9)
Vitality	42 (11)	33 (10)	39 (10)
Mental health	46 (11)	40 (11)	45 (11)
PCS	29 (12)	16 (8)	24 (11)
MCS	47 (12)	44 (11)	48 (11)
DMARD by 6-month follow-up	91%	100%	100%
First recorded DMARD			
MTX monotherapy	48%	40%	0%
SSZ monotherapy	31%	14%	0%
MTX combination	12%	25%	0%
Biologic	0%	0%	100%

Baseline variables of the complete cases for the cohorts. Mean (SD) or percentage data are presented

*Abbreviations: ACR* American College of Rheumatology, *BSRBR* British Society for Rheumatology Biologics Registers, *DAS28* 28-joint Disease Activity Score, *DMARD* Disease-modifying anti-rheumatic drug, *ERAN* Early Rheumatoid Arthritis Network, *ESR* Erythrocyte sedimentation rate, *HAQ* Health Assessment Questionnaire, *SF-36* 36-item Short Form Health Survey, *SJC* Swollen joint count, *TJC* Tender joint count, *TNF* Tumour necrosis factor, *VAS* Visual analogue scale, *PCS* Short Form Health Survey physical component score, *MCS* Short Form Health Survey mental component score, *MTX* methotrexate, *SSZ* sulphasalazine

### Selection of variables

Variables were selected for LCA on the basis of our a priori hypothesis if they measured factors believed to reflect current inflammation, pain experience or central pain processing. Three SF-36 subscales relevant to our hypothesis were included. Bodily pain is a direct measure of pain symptoms and functional limitation attributed to pain [33]. Vitality is a measure of fatigue, and SF-36 mental health component scores use items addressing low mood and anxiety. Additional variables were used to compare baseline demographic and clinical characteristics between latent classes (age, sex, current smoking status, symptom/disease duration, body mass index, serology, SF-36 mental and physical component scores, or DAS28). Clinically relevant outcome variables (DAS28, SF-36 physical function score and HAQ) [34] were also retrieved from baseline to 1-year follow-up.

### Statistical analysis

LCAs were performed on baseline data. Variables were standardised prior to LCA using their theoretical maximum so that each had a range of 0–1 and had higher values indicating greater severity. SF-36 subscales were transformed using the formula (100 − SF-36)/100, which yielded standardised scores of increasing severity, and ESR values were log-transformed. List-wise exclusion of missing data (complete case analysis) was employed as our primary analysis strategy. Missingness was highest for SF-36 variables and ESR, which were between 10% and 20%, and other variables were all below 5%.

The selection of the optimum number of classes was guided by iteratively comparing several diagnostic indices [35, 36] from *k* categories to those from the *k* − 1 categories. Lower values of the Bayesian information criterion, the Akaike information criterion and a non-significant Vuong-Lo-Mendell-Rubin (VLMR) adjusted likelihood ratio test, as well as the bootstrap likelihood ratio test (BLRT), all suggested better fit. Higher values of the entropy implied higher probabilities of people being assigned to the correct latent class. The VLMR and the BLRT both had associated significance tests for guidance purposes, whereas the other measures were assessed by comparison with their scores with *k* − 1 categories. Models where latent classes showed membership of <5% of the total cohort size were rejected. Thus, the final determination of classes was based on theoretical and clinical significance in conjunction with the fit indicators [37].

Descriptive names for different classes were selected to reflect our hypothesis by comparing the discordance or concordance of values of patient-reported measures with clinician- or laboratory-assessed measures. Clinical judgement and the relative values of each score (both within and across cohorts) were taken into account. These were selected after the LCA had been completed and the mean values of each class were known. Characteristics of participants allocated to each class were compared using one-way analysis of variance (ANOVA) and then using pairwise *t* tests with Bonferroni corrections. LCA was performed using Mplus version 7.2 software (Muthén and Muthén, Los Angeles, CA, USA). Data management and analyses other than LCA were performed using IBM SPSS version 22 software (IBM, Armonk, NY, USA). Statistical significance was taken as *p* < 0.05.

DAS28, HAQ and SF-36 physical function outcomes during the first year of follow-up were assessed graphically and using descriptive statistics for change values for each latent class, and by comparing 12-month follow-up data between latent classes using multivariate ANOVA. Only participants with complete data at three time points (baseline, 3–6 months, and 12-month follow-up) were included, and the demographics and clinical characteristics of this subgroup were compared with those included in baseline analyses to ensure appropriate representation.

## Results

Baseline demographics and clinical details of participants included for LCA in each cohort are presented in Table 1. ERAN participants had shorter symptom duration (median 0.5 years) than BSRBR participants (median ≥6 years) and were less likely than BSRBR participants to satisfy 1987 American College of Rheumatology criteria for RA (53% vs. 100%, respectively). BSRBR TNF inhibitor participants had higher DAS28 scores, worse HAQ disability and worse quality-of-life measures than did ERAN and BSRBR non-biologic participants.

**Fig. 1:**
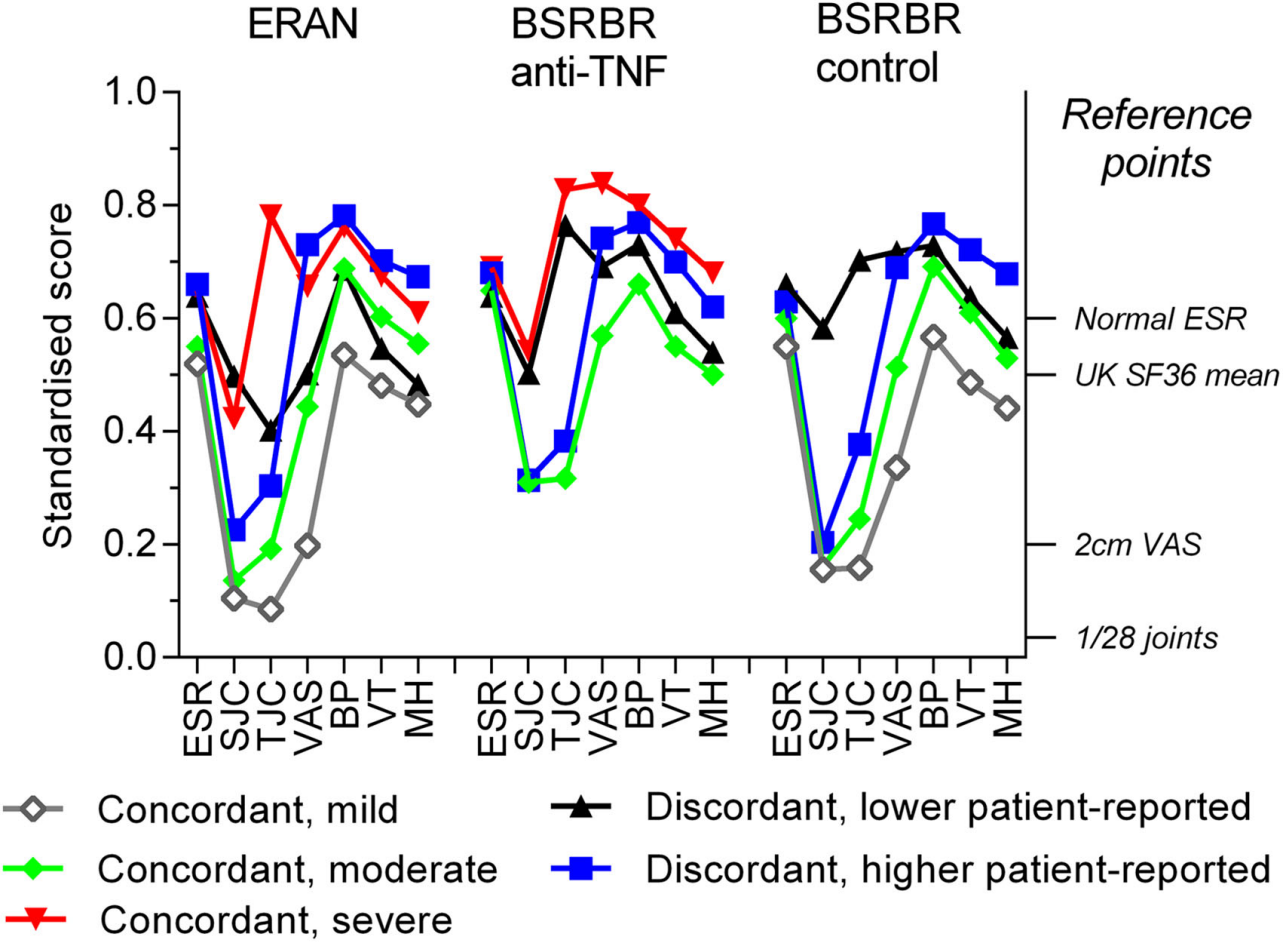
Latent classes of baseline inflammation and pain-related variables. *Left y*-axis = variables standardised from 0 to 1 to allow comparison between different indices. Increasing severity is reflected by increasing scores. *Right y*-axis = reference points for normal or low activity scores for different variables. *BP* Short Form Health Survey bodily pain score, *BSRBR* British Society for Rheumatology Biologics Registers, *ERAN* Early Rheumatoid Arthritis Network, *VT* Short Form Health Survey vitality score, *MH* Short Form Health Survey mental health score, *ESR* Erythrocyte sedimentation rate, *SF-36* 36-item Short Form Health Survey, *SJC* Swollen joint count, *TJC* Tender joint count, *TNF* Tumour necrosis factor *VAS* Visual analogue scale. Names of classes are descriptive

Comparable latent classes were identified in each of the three cohorts. The process of selecting LCA models for each cohort is summarised through the indices listed in Table 2. When all of the different diagnostic indices were taken into account, the final decision regarding the number of classes was taken in reference to two main factors: the size of the latent classes and VLMR *p* values reaching cut-off criteria. Further selection of additional latent classes would be likely to be less robust. The composition of each latent class is summarised in Table 3, showing the population percentage, the mean scores for each variable, and the percentage of cases that the model classified with higher levels of likelihood (80% or 95% probabilities). Five latent classes were selected in the ERAN cohort, and four were selected in the BSRBR cohorts (Fig. 1). Latent classes that had been selected were named on the basis of clinical interpretation of group characteristics, severity (mild, moderate, severe) and concordance of patient-reported with clinician-observed and laboratory-measured variables.

**Table 2:** Summary of selection process for latent class analysis models

Classes in model	AIC	BIC	SS-BIC	Entropy	VLMR	BLRT	Percentage in each class (in order of size)					
							Class 1	Class 2	Class 3	Class 4	Class 5	Class 6
ERAN cohort												
1	−5458	−5392	−5738	Not available			100%					
2	−5858	−5753	−5823	0.78	2387^*^	2387^*^	56%	44%				
3	−6229	−6087	−6182	0.81	2951^*^	2951^*^	52%	33%	15%			
4	−6395	−6214	−6335	0.80	3145^**^	3145^*^	48%	27%	13%	12%		
**5**	**−6552**	**−6333**	**−6480**	**0.80**	**3235** ^***^	**3235** ^*^	**40%**	**26%**	**13%**	**11%**	**10%**	
6	−6645	−6389	−6560	0.82	3311	3311^*^	40%	26%	11%	11%	10%	2%
British Society for Rheumatology Biologics Registers TNF inhibitor cohort												
1	−69,795	−69,695	−69,739	Not available			100%					
2	−77,781	−77,623	−77,693	0.65	36,175^*^	36,175^*^	62%	38%				
3	−79,445	−79,229	−79,325	0.71	38,913^**^	38,913^*^	53%	41%	6%			
**4**	**−80,851**	**−80,578**	**−80,698**	**0.69**	**39,753** ^*^	**39,753** ^*^	**40%**	**23%**	**21%**	**16%**		
5	−81,896	−81,565	−81,711	0.70	40,464^*^	40,464^*^	32%	24%	22%	19%	2%	
British Society for Rheumatology Biologics Registers non-biologic cohort												
1	−19,209	−19,127	−19,172	Not available			100%					
2	−21,497	−21,368	−21,438	0.70	9618^*^	9618^*^	56%	44%				
3	−22,638	−22,462	−22,557	0.76	10,770^*^	10,770^*^	51%	34%	15%			
**4**	**−23,170**	**−22,947**	**−23,068**	**0.76**	**11,349** ^*^	**11,349** ^*^	**51%**	**21%**	**18%**	**10%**		
5	−23,457	−23,188	−23,334	0.76	11,623	11,623^*^	45%	20%	19%	11%	5%	

Selection diagnostics for latent class analysis are summarised for models with different numbers of classes. Each model was compared with the previous model with one fewer latent classes. The percentage assigned to each latent class is shown. The model that was selected as the optimal fit is highlighted in bold

*Abbreviations: AIC* Akaike information criterion, *BIC* Bayesian information criterion, *BLRT* Bootstrap likelihood ratio test, *ERAN* Early Rheumatoid Arthritis Network, *TNF* Tumour necrosis factor, *VLMR* Vuong-Lo-Mendell-Rubin likelihood ratio test, *SS-BIC* Sample size-adjusted Bayesian information criterion

^*^ *p* < 0.001

^**^ *p* < 0.01

^***^ *p* < 0.05 (significance tests are applied for VLMR and BLRT only)

**Table 3:** Composition of each latent class

	ERAN					BSRBR TNF inhibitor cohort				BSRBR non-biologic cohort			
Concordant/discordant	Con	Con	Con	Dis	Dis	Con	Con	Dis	Dis	Con	Con	Dis	Dis
Descriptor	Mild	Moderate	Severe	Better patient-reported	Worse patient-reported	Moderate	Severe	Better patient-reported	Worse patient-reported	Mild	Moderate	Better patient-reported	Worse patient-reported
Number of subjects	220	329	86	91	102	1544	2117	2289	3955	476	1308	248	549
Percentage of total	26%	38%	10%	11%	12%	16%	21%	23%	40%	18%	51%	10%	21%
ESR	22 (19)	25 (18)	42 (29)	40 (26)	44 (29)	42 (27)	52 (31)	39 (26)	49 (29)	27 (21)	33 (23)	44 (28)	38 (26)
SJC	3 (3)	4 (3)	12 (6)	14 (5)	7 (4)	9 (5)	15 (6)	14 (6)	9 (5)	4 (4)	5 (3)	16 (5)	6 (4)
TJC	2 (3)	5 (4)	22 (4)	11 (5)	9 (5)	9 (4)	23 (4)	21 (4)	11 (4)	4 (4)	7 (5)	20 (6)	11 (6)
VAS	20 (15)	44 (20)	66 (20)	50 (20)	73 (16)	57 (20)	84 (15)	69 (19)	74 (18)	34 (21)	51 (21)	72 (18)	69 (16)
BP	46 (6)	31 (7)	24 (6)	32 (6)	22 (5)	34 (7)	20 (4)	27 (6)	23 (5)	43 (6)	31 (5)	27 (7)	23 (5)
VT	52 (9)	40 (8)	33 (8)	46 (8)	30 (8)	45 (9)	25 (6)	39 (9)	30 (7)	51 (7)	39 (7)	36 (9)	28 (6)
MH	55 (7)	45 (9)	39 (9)	52 (8)	32 (10)	50 (9)	32 (10)	46 (9)	38 (10)	56 (7)	47 (9)	43 (10)	32 (9)
Probability of membership of latent class													
> 80% probability	89%	73%	78%	57%	70%	66%	65%	53%	66%	74%	74%	77%	63%
> 95% probability	71%	40%	56%	30%	41%	40%	37%	22%	38%	51%	36%	59%	38%

Baseline variables used in latent class analysis are summarised to describe composition of each latent class identified within each cohort. The number of participants and the mean (SD) of each variable are shown. The percentage of cases allocated to each latent class is shown for those with a probability of membership in that class >80% or >95%

*Abbreviations: BSRBR* British Society for Rheumatology Biologics Registers, *Dis* Discordant, *Con* Concordant, *ESR* Erythrocyte sedimentation rate, *SJC* Swollen joint count, *TJC* Tender joint count, *VAS* Visual analogue scale for general health, *BP* Short Form Health Survey bodily pain, *VT* Short Form Health Survey vitality, *MH* Short Form Health Survey mental health, *TNF* Tumour necrosis factor

In each cohort, at least two latent classes displayed concordance between patient-reported, clinician-observed and laboratory-measured indices. These latent classes were distinguished by severity in each scale. Each cohort also included one discordant latent class with worse patient-reported indices, but relatively low clinician-observed and laboratory-measured indices. Furthermore, each cohort also included one latent class with discordantly low patient-reported indices, despite relatively high clinician-observed or laboratory-measured indices.

In the ERAN cohort, three concordant classes appeared to reflect, respectively, subgroups with mild (26% of participants), moderate (38%) or severe RA (10%) (Table 3, Fig. 1). The discordant latent class in ERAN with worse patient-reported indices (12%) showed the worst mean bodily pain, VAS-GH, vitality and mental health scores alongside laboratory-measured and physician-observed indices that were comparable (ESR) or better (SJC) in the concordant severe latent class. The discordant latent class in ERAN with better patient-reported indices (11%) showed high levels of the laboratory-measured and physician-observed indices (ESR, SJC), which were comparable to the concordant, severe latent class, alongside better scores for patient-reported indices (comparable to the moderate or mild, concordant latent classes). Concordant moderate and mild or severe latent classes, and discordant with worse or better patient-reported indices, were identified in the BSRBR cohorts that were comparable to those identified in ERAN. Discordant latent classes with relatively worse patient-reported measures comprised 12%, 40% and 21% of ERAN, BSRBR TNF inhibitor and non-biologic participants, respectively (Table 3), and displayed age, sex, DAS28 and HAQ scores similar to the concordant severe classes (Table 4). Comparison of additional baseline characteristics between latent classes is given in Additional file 1: Table S1.

**Table 4:** Baseline characteristics of the latent classes

	ERAN						BSRBR TNF inhibitor cohort					BSRBR control cohort				
Concordant/Discordant	Con	Con	Con	Dis	Dis	Heterogeneity	Con	Con	Dis	Dis	Heterogeneity	Con	Con	Dis	Dis	Heterogeneity
Descriptor	Mild	Moderate	Severe	Better patient-reported	Worse patient-reported		Moderate	Severe	Better patient-reported	Worse patient-reported		Mild	Moderate	Better patient-reported	Worse patient-reported	
Age	58 (13)	56 (14)	55 (14)	58 (13)	57 (13)		56 (13)	56 (12)	57 (14)	56 (12)	*	60 (12)	61 912)	59 (12)	59 (12)	*
Female	60%	68%	65%	66%	81%	*	72%	79%	75%	77%	*	69%	73%	71%	77%	*
DAS28	3.2 (1.1)	4.3 (1.0)	6.9 (0.8)	6.0 (0.7)	5.7 (0.8)	*	5.6 (0.9)	7.5 (0.7)	7.0 (0.7)	6.2 (0.7)	*	4.1 (1.1)	4.9 (1.0)	7.0 (0.8)	5.7 (1.0)	*
HAQ	0.4 (0.5)	1.1 (0.6)	1.8 (0.6)	1.3 (0.6)	1.8 (0.7)	*	2.0 (0.6)	2.0 (0.6)	2.1 (0.6)	2.0 (0.6)		0.7 (0.6)	1.5 (0.6)	1.8 (0.6)	2.1 (0.5)	*

Clinical variables not used in the latent class analysis are compared between identified latent classes. Mean (SD) or percentage data are presented

*Abbreviations: BSRBR*, British Society for Rheumatology Biologics Registers, *DAS28* 28-joint Disease Activity Score, *ERAN* Early Rheumatoid Arthritis Network, *HAQ* Health Assessment Questionnaire, *TNF* Tumour necrosis factor, *Dis* Discordant, *Con* Concordant

*p < 0.05 from one-way analysis of variance for heterogeneity between latent classes within one cohort

**Fig. 2:**
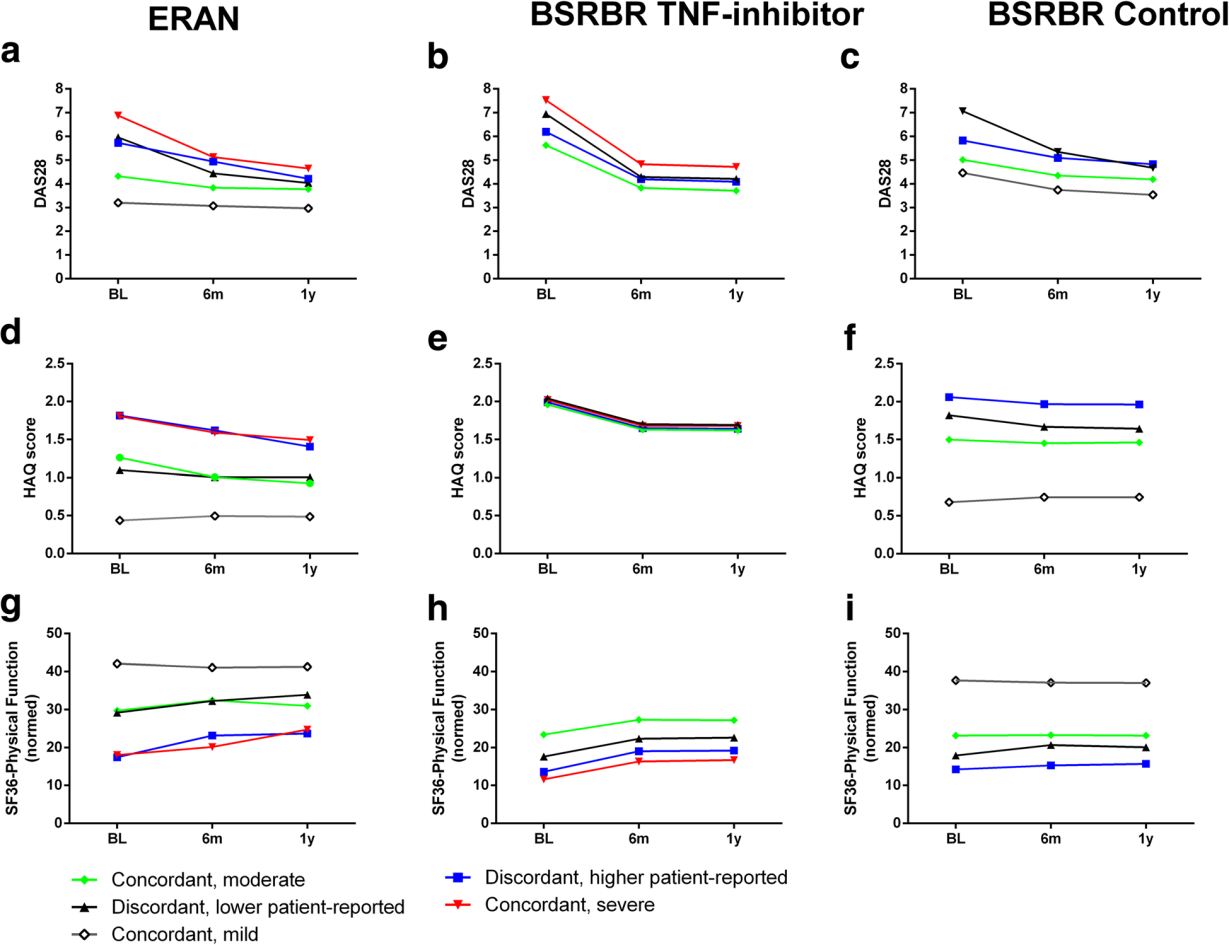
Early longitudinal changes in the three cohorts. Changes in mean clinical and functional scores over the first year from recruitment for 28-joint Disease Activity Score (DAS28) (**a**, **b**, **c**), Health Assessment Questionnaire (HAQ) (**d**, **e**, **f**) and 36-item Short Form Health Survey (SF-36) physical function (**g**, **h**, **i**), stratified by latent class subgroup. Cohorts displayed are the Early Rheumatoid Arthritis Network (ERAN) (**a**, **d**, **g**), British Society for Rheumatology Biologics Registers (BSRBR) tumour necrosis factor (TNF) inhibitor (**b**, **e**, **h**) and BSRBR non-biologic (**c**, **f**, **i**). *BL* Baseline, *6 m* 6 months, *1y* 1 year. Complete cases were analysed, yielding respective sample sizes for ERAN, BSRBR TNF inhibitor and BSRBR non-biologic for DAS28 (*n* = 383, 6376 and 887, respectively), HAQ (*n* = 447, 5627 and 1601, respectively) and SF-36 physical function (*n* = 217, 6179 and 1706, respectively). There was significant heterogeneity between the changes in scores of all of the latent classes for each cohort and for each measure (*p* < 0.001 for each comparison)

Mean DAS28 and functional status scores changed at different rates during 12 months of follow-up (*p* < 0.001 for all comparisons) (Fig. 2a–c) and improved most in those latent classes with the worst baseline scores (Fig. 2). Baseline scores explained changes in HAQ across 12 months because the multivariate test of different latent classes lost significance after adjustment for baseline HAQ in each cohort (*p* = 0.374, *p* = 0.772 and *p* = 0.238 for ERAN, BSRBR biologic and non-biologic cohorts, respectively). The changes in DAS28 across 12 months were not completely explained by baseline DAS28, because the significance of the multivariate test for the different latent classes was retained (*p* = 0.026, *p* < 0.001 and *p* < 0.0.001, respectively), and changes in SF-36 physical function were explained only by baseline scores in one cohort (*p* = 0.080, *p* < 0.001 and *p* < 0.001, respectively).

## Discussion

We have shown that people with RA can be allocated to four or five discrete latent classes that reflect either differing degrees of inflammatory disease activity or discordance between patient-reported and clinician- or laboratory-measured indices of inflammation. Our analyses show subgroups of patients with RA for whom DAS28 might either underestimate (discordant, worse patient-reported indices in 12–40% of participants) or overestimate (discordant, better patient-reported indices in 10–23%) their need for DMARD escalation or for whom additional strategies might be used to augment clinical benefit from biologic or non-biologic DMARDs.

DAS28 is widely used to direct RA treatment with biologic or non-biologic treatments. Some countries restrict access to biologic therapy to those patients who have a persistent DAS28 above a threshold level [38], and treat-to-target approaches are increasingly recommended whereby DAS28 informs DMARD treatment escalation [17]. We have consistently identified discordant subgroups within all three studied cohorts, suggesting generalisability through both early and established RA and in patients with apparently active disease being considered for either biologic or non-biologic DMARDs. Discordant subgroups represented 23–63% of participants in each studied cohort, suggesting that non-inflammatory factors have a major influence on interpretation of DAS28 as a measure of inflammatory disease activity.

Central mechanisms might augment pain in people with RA, and this discordance between DAS28 components might reflect the balance between peripherally and centrally acting pain mechanisms [3, 7–9, 38]. Our data are therefore consistent with previous findings that pain moderates inflammatory disease assessment [5, 7, 11, 14] and that DAS28 might overestimate inflammatory disease activity, particularly in the context of augmented central pain processing [32].

In addition, we identified discrete subgroups of patients for whom patient-reported measures were better than might be expected from clinician- and laboratory-measured DAS28 components. The ‘typus robustus’ RA phenotype [39] might represent one extreme of this discordant subgroup. It is increasingly recognised that patients with moderate disease activity based on DAS28 have poor outcomes, and this might particularly be the case for those in whom total DAS28 is suppressed by discordantly low patient-reported components. This might reflect a hidden need for escalating biologic or non-biologic DMARD therapy. However, further research is needed to determine whether DMARD escalation in this patient subgroup would have greater benefits for retarding joint damage and chronic disability than in people with similar DAS28 who are concordant or who show discordantly high patient-reported measures.

Our data support previous observations that people with more severe disease at baseline, measured either by patient self-report or by clinician or laboratory assessment, appear to have the greatest scope for improvements, and latent classes with worse baseline scores displayed greater improvements during follow-up. Improvement in those with worse baseline scores might partially be attributable to regression to the mean, but might also be expected after commencing new treatments. Further research is required to determine whether patients with discordantly high patient-reported indices might gain particular benefit from adjunctive treatments targeting pain management and psychological distress. Despite the prospective nature of our study cohorts, we are unable to distinguish between sustained disease activity and shorter-term flares. Further research might investigate whether latent classes defined by inflammation and patient-reported variables can predict inflammatory or symptomatic flares in people with RA.

DMARDs improve patient-reported indices by suppressing inflammation and through placebo effects [40], which might be particularly pronounced for expensive treatments with limited availability, such as biologic agents. Furthermore, inflammation might drive changes in central neuronal processes, leading to augmented pain, fatigue and mood disturbance [41]. These multiple peripheral and central modes of action might conceal treatment response differences between latent classes, despite differing underlying disease mechanisms. We did not identify differences in responses to biologic or non-biologic DMARDs between discordant patient subgroups. Nonetheless, our data indicate that patient subgroups can be identified prior to treatment changes on the basis of existing, easily administered clinical assessment and questionnaires. Entropy measurements in the 0.7–0.8 range for all models tested in the present study indicate good levels of confidence in our subgrouping of individuals.

Patient-reported variables might be particularly useful for stratifying treatment modalities used as adjuncts to DMARD therapies. However, using widespread pain alone for selective recruitment of participants was not adequate in a clinical trial of the centrally acting analgesic milnacipran in people with RA, although the number of participants might be small and secondary analysis implicated a confounding effect of synovitis on the pain measurements [42]. Clustering algorithms based on studies such as ours and others [19] represent an alternative tool for determining recruitment criteria for personalised medicine trials.

There are some limits to the generalisability of our findings. The characteristics of each cohort will have influenced the latent classes that were identified. For example, a ‘concordant, mild’ subgroup was not identified in the BSRBR TNF inhibitor cohort, probably because patients were recruited to this cohort following selection for TNF inhibitor treatment based on the presence of active inflammatory disease. DAS28 scores were measured by different assessors across different centres, and variability in scores between cohorts and between study centres might have introduced bias, as well as lowering precision. It is possible that the non-independent assessments of SJC and TJC (assessed during a single clinical examination) might also have introduced bias and reduced apparent discordance between these patient-reported and clinician-assessed components. Patients in the BSRBR non-biologic cohort were also recruited with clinician-assessed active disease, and patients were recruited to ERAN shortly after first presentation to secondary care, usually before DMARD therapy had been optimised. Subgroups with RA and mild inflammatory disease activity or discordantly low DAS28 components might therefore be under-represented in our cohorts compared with a more general RA population. Furthermore, most participants in our study changed DMARD therapy after baseline assessment, and different latent classes might be found in people with RA undergoing stable treatment. However, the consistency of our findings across different RA populations, as well as evidence of central sensitisation or from classification of fibromyalgia in established stable RA [7, 8, 10], suggests that our findings might be generally applicable. The relationship between latent classes identified in this study and underlying disease mechanisms requires further elucidation, and more direct measures of central pain mechanisms (e.g., quantitative sensory testing or functional magnetic resonance imaging) might better distinguish between patient subgroups.

## Conclusions

We demonstrate latent classes amongst patients with early or established RA based on patient-reported and clinician- or laboratory-measured indices of inflammation and pain. Discordant classes representing patients with worse or better patient-reported measures than might be suggested by clinician- or laboratory-measured inflammation contribute a high proportion of people with high DAS28. Randomised controlled trials are needed to determine whether patient subgrouping or stratification based on patient-reported and clinician- or laboratory-measured indices can improve effectiveness or cost-effectiveness of RA management or reduce unnecessary exposure of patients to DMARD and biologic escalation strategies when interventions targeting pain processing might be more suitable.

## Additional file

Additional file 1: Table S1. Baseline characteristics of each latent class (including data from Table 4). (DOCX 18 kb)

## Abbreviations

ACR: American College of Rheumatology
AIC: Akaike information criterion
ANOVA: Analysis of variance
BIC: Bayesian information criterion
BLRT: Bootstrap likelihood ratio test
BMI: Body mass index
BP: Short Form Health Survey bodily pain
BSRBR: British Society for Rheumatology Biologics Registers
Con: Concordant
DAS28: 28-joint Disease Activity Score
Dis: Discordant
DMARD: Disease-modifying anti-rheumatic drug
ERAN: Early Rheumatoid Arthritis Network
ESR: Erythrocyte sedimentation rate
HAQ: Health Assessment Questionnaire
LCA: Latent class analysis
MCS: Short Form Health Survey mental component score
MH: Short Form Health Survey mental health
MTX: methotrexate
PCS: Short Form Health Survey physical component score
SF-36: 36-item Short Form Health Survey
SJC: Swollen joint count
SS-BIC: Sample size-adjusted Bayesian information criterion
SSZ: sulphasalazine
TJC: Tender joint count
TNF: Tumour necrosis factor
VAS: Visual analogue scale
VAS-GH: Visual analogue scale for general health
VLMR: Vuong-Lo-Mendell-Rubin likelihood ratio test
VT: Short Form Health Survey vitality
