# Supplementary material for: Discordant inflammation and pain in early and established rheumatoid arthritis: Latent Class Analysis of Early Rheumatoid Arthritis Network and British Society for Rheumatology Biologics Register data
Source: Arthritis Res Ther. 2016 Dec 13;18:295. doi: 10.1186/s13075-016-1186-8 (PMC5154167; doi:10.1186/s13075-016-1186-8)
Supplement: Additional file 1: Table S1. — Baseline characteristics of each latent class (including data from Table 4). (DOCX 18 kb) [file 13075_2016_1186_MOESM1_ESM.docx]

Additional file 1

Table S1: Baseline characteristics of each latent class (including data from Table 4)

|  | ERAN | | | | |  |  | TNF-inhibitor | | | |  |  | Control | | | |  |
| --- | --- | --- | --- | --- | --- | --- | --- | --- | --- | --- | --- | --- | --- | --- | --- | --- | --- | --- |
| Concordant / Discordant | Con | Con | Con | Dis | Dis | Heterogeneity |  | Con | Con | Dis | Dis | Heterogeneity |  | Con | Con | Dis | Dis | Heterogeneity |
| Descriptor | Mild | Moderate | Severe | Lower patient-reported | Higher patient-reported |  |  | Moderate | Severe | Lower patient-reported | Higher patient-reported |  |  | Mild | Moderate | Lower patient-reported | Higher patient-reported |  |
| Age | 58 (13) | 56 (14) | 55 (14) | 58 (13) | 57 (13) |  |  | 56 (13) | 56 (12) | 57 (14) | 56 (12) | * |  | 60 (12) | 61 912) | 59 (12) | 59 (12) | * |
| Female | 60% | 68% | 65% | 66% | 81% | * |  | 72% | 79% | 75% | 77% | * |  | 69% | 73% | 71% | 77% | * |
| BMI | 27 (5) | 28 (5) | 28 (9) | 28 (5) | 28 (6) |  |  | 26 (5) | 27 (7) | 27 (8) | 27 (8) | * |  | 27 (6) | 27 (7) | 28 (6) | 28 (6) | * |
| Current smoker | 35% | 38% | 36% | 35% | 26% |  |  | 18% | 23% | 19% | 24% | * |  | 17% | 21% | 23% | 25% | * |
| Seropositive | 58% | 57% | 70% | 61% | 64% |  |  | 64% | 68% | 67% | 63% | * |  | 56% | 59% | 47% | 56% | * |
| ACR | 31% | 52% | 80% | 73% | 63% | * |  | 100% | 100% | 100% | 100% |  |  | 100% | 100% | 100% | 100% |  |
| Duration (yr) | 0.8 (0.6) | 0.7 (0.5) | 0.8 (0.8) | 0.7 (0.5) | 0.6 (0.6) |  |  | 13 (10) | 13 (10) | 13 (10) | 13 (10) |  |  | 9 (10) | 10 (10) | 8 (10) | 10 (10) | * |
| DAS28 | 3.2 (1.1) | 4.3 (1.0) | 6.9 (0.8) | 6.0 (0.7) | 5.7 (0.8) | * |  | 5.6 (0.9) | 7.5 (0.7) | 7.0 (0.7) | 6.2 (0.7) | * |  | 4.1 (1.1) | 4.9 (1.0) | 7.0 (0.8) | 5.7 (1.0) | * |
| HAQ | 0.4 (0.5) | 1.1 (0.6) | 1.8 (0.6) | 1.3 (0.6) | 1.8 (0.7) | * |  | 2.0 (0.6) | 2.0 (0.6) | 2.1 (0.6) | 2.0 (0.6) |  |  | 0.7 (0.6) | 1.5 (0.6) | 1.8 (0.6) | 2.1 (0.5) | * |
| PF | 42 (11) | 30 (12) | 18 (12) | 30 (12) | 18 (12) | * |  | 23 (12) | 11 (9) | 17 (10) | 14 (9) | * |  | 38 (12) | 23 (12) | 18 (11) | 14 (10) | * |
| PCS | 41 (9) | 28 (10) | 18 (8) | 25 (10) | 19 (8) | * |  | 23 (10) | 13 (6) | 17 (9) | 15 (7) | * |  | 37 (10) | 23 (10) | 18 (9) | 17 (8) | * |
| MCS | 56 (8) | 45 (9) | 38 (10) | 54 (9) | 34 (9) | * |  | 55 (9) | 35 (9) | 50 (9) | 41 (10) | * |  | 59 (7) | 50 (9) | 46 (10) | 36 (8) | * |
| DAS28-P | 0.32 (0.11) | 0.42 (0.10) | 0.52 (0.05) | 0.42 (0.08) | 0.45 (0.08) | * |  | 0.42 (0.08) | 0.52 (0.05) | 0.51 (0.06) | 0.46 (0.07) | * |  | 0.38 (0.11) | 0.43 (0.10) | 0.49 (0.06) | 0.47 (0.08) | * |

Clinical variables not used in the LCA are compared between identified latent classes. Mean (sd) or percent are presented. Heterogeneity (p<0.05 from one-way ANOVA) between latent classes within one cohort denoted by *.
